# S08-1 Comparing physical activity measurement instruments across member states by means of a conversion factor: European Union Physical Activity and Sport Monitoring System (EUPASMOS) project

**DOI:** 10.1093/eurpub/ckac093.040

**Published:** 2022-08-29

**Authors:** Ellen de Hollander

**Affiliations:** National Institute for Public Health and the Environment, Bilthoven, The Netherlands

**Keywords:** sedentary behavior, questionnaire, accelerometer, conversion factor, Europe

## Abstract

**Background:**

The successful promotion of health-enhancing physical activity (HEPA) requires authoritative information to enable the design, implementation and evaluation of effective and cost-effective policies. This includes reliable and valid prevalence data of physical activity (PA) that can be compared across European Union Member States populations regardless of the type of measurement instrument used. In order to enable comparison of PA prevalence data between different measurement instruments, an attempt was made to develop a conversion factor between commonly used questionnaires in Europe and accelerometry in the ongoing European Union Physical Activity and Sport Monitoring System project (EUPASMOS) among 18 member states.

**Methods:**

Data of physical activity (PA) and sedentary behavior (SB) were collected in 18 member states using four questionnaires (Eurobarometer, European Health Interview Survey, International Physical Activity Questionnaire and Global Physical Activity Questionnaire) and the UKK RM42 accelerometer. The number of measured participants varied between 100 and 1000 among the 18 member states. Participants completed the questionnaires in random order and wore the accelerometer seven consecutive days on their right hip (during the day) and on their non-dominant wrist (overnight). Time spent in moderate-to-vigorous intensity physical activity and sedentary behavior were calculated based on these 5 measurement instruments. The comparison between methods will be examined by using sophisticated regression analyses.

**Results:**

The EUPASMOS project is in progress and the final data is collected in the last two member states. In the meantime, a model is being developed to examine whether a conversion factor can be calculated to compare prevalence data based on questionnaires and accelerometry. By developing a conversion factor, prevalence data can be compared between member states although the measurement instruments used differ between member states. In June 2020 the results from these analyses are expected.

**Conclusions:**

These results will provide insight into the possibility to compare prevalence data of physical activity among member states, when using different measurement instruments within their monitoring systems.

